# Knee and Hip Muscle Strength of Male Soccer Players from Different Competitive Levels

**DOI:** 10.5114/jhk/185217

**Published:** 2024-05-17

**Authors:** Cíntia França, Francisco Martins, Krzysztof Przednowek, Adilson Marques, Andreas Ihle, Hugo Sarmento, Élvio Rúbio Gouveia

**Affiliations:** 1 Department of Physical Education and Sport, University of Madeira, Funchal, Portugal; 2 LARSYS, Interactive Technologies Institute, Funchal, Portugal; 3 Institute of Physical Culture Sciences, Medical College, University of Rzeszów, Rzeszów, Poland; 4 CIPER, Faculty of Human Kinetics, University of Lisbon, Lisbon, Portugal; 5 ISAMB, Faculty of Medicine, University of Lisbon, Lisbon, Portugal; 6 Department of Psychology, University of Geneva, Geneva, Switzerland; 7 Center for the Interdisciplinary Study of Gerontology and Vulnerability, University of Geneva, Geneva, Switzerland; 8 Swiss Centre of Expertise in Life Course Research LIVES, Geneva, Switzerland; 9 Research Unit for Sport and Physical Education (CIDAF), Faculty of Sport Sciences and Physical Education, University of Coimbra, Coimbra, Portugal.

**Keywords:** football, isokinetic strength, knee flexors, knee extensors, squeeze strength

## Abstract

In soccer, knee and hip muscle strength assessments have been recommended for injury prevention. The aims of this study were threefold: (1) to compare knee and hip muscle strength between professional players competing at different levels; (2) to compare strength performance according to the preferred leg (PL) and the non-preferred leg (NPL); and (3) to compare knee and hip muscle strength performance at two moments of the season. This study included 33 professional soccer players: 13 were in the elite group (EG), and 20 were in the sub-elite group (SEG). Body composition, isokinetic knee strength at 60º/s, and hip adduction strength were assessed at two different moments (M1 and M2). Values of peak torque (PT), peak torque/bodyweight (PT/BW), and the hamstring-to-quadriceps strength ratio (H:Q) for knee extensors (KEs) and knee flexors (KFs) for both legs were used for analysis. The statistical analysis included the Mann-Whitney U and the Wilcoxon Signed Rank tests. At M1, the EG presented a significantly better performance in KF PT/BW and in the squeeze strength test for the PL and the NPL (p ≤ 0.01). At M2, the EG performed substantially better in KE PT/BW and KF PT/BW (p ≤ 0.01). No substantial strength differences were observed in knee and hip muscle performance between the PL and the NPL. From M1 to M2, significant increases were found in knee strength in both groups (p ≤ 0.01). Overall, the EG players outperformed significantly their lower-division peers in strength assessments. The results indicate significant knee and hip muscle strength increases during the season, probably as a response to the exposure to training and competition.

## Introduction

Playing high-level soccer imposes increased physical demands to tackle high-speed and intense actions (Chena et al., 2022; Konefał et al., 2023). Performing high-intensity efforts may increase the risk of injury, resulting in players’ absences from subsequent training sessions or competitions (Hägglund et al., 2005). Previous research has reported that high-intensity horizontal decelerations can lead to tissue damage and neuromuscular fatigue, putting players at the risk of injury (McBurnie et al., 2022). Additionally, a high number of intense high-speed actions performed in soccer, requires the hamstrings to perform in a position of an extreme stretch, which may cause injury (Fernández-Baeza et al., 2022). According to the sports literature, performance of high-intensity movements, such as jumping, sprinting, and change of direction tasks, has been strongly associated with the rate of force development and, consequently, with muscle strength (Stone et al., 2002). Overall, research indicates that greater muscle strength can enhance match performance and decrease the risk of injury, which underlines strength as one of the most crucial attributes for soccer players (Suchomel et al., 2016).

Previous research on professional male soccer players reported the quadriceps as the most affected body zone by injury, followed by the hamstrings and adductors (Martins et al., 2022). Although the results may vary in several investigations, the literature has consistently described those muscles as the most affected by injury in frequency and severity (López-Valenciano et al., 2020). In soccer, muscle imbalances for knee extensors (KEs) and knee flexors (KFs) have been linked to hamstring strain (Croisier et al., 2008; Krolikowska et al., 2023;Lord et al., 2018). Of 687 soccer players isokinetically tested in a previous study, the results showed that hamstring injury was significantly increased (relative risk index of 4.66) in players with untreated strength imbalances when compared to players showing no imbalance in the preseason (Croisier et al., 2008). In another investigation among 20 male soccer players, authors reported that decreases in peak KF and KE torque during an isokinetic endurance test after repeated sprint exercise were able to identify previous hamstring injuries (Lord et al., 2018). According to the literature, strength imbalances are related to a significant deficit in at least one of the following variables: concentric bilateral imbalance, eccentric bilateral imbalance, concentric flexors/quadriceps ratio, and mixed eccentric flexors/concentric quadriceps ratio (Croisier et al., 2002). Bilateral differences of 15% or more, a concentric ratio of less than 0.47, and a mixed ratio of less than 0.80 based on isokinetic variables may be considered severe (Croisier et al., 2002; Fischerova et al., 2021; Pfirrmann et al., 2016).

Concerning injury occurrence, the weakness of hip adduction has also been identified as a risk factor for groin injury (Engebretsen et al., 2010; Ryan et al., 2014). In a sample of 508 male amateur soccer players, authors stated that previous acute groin injury and weak adductor muscles were significant risk factors for new groin injuries (Engebretsen et al., 2010). The same conclusion was presented in a systematic review of the risk factors for groin injuries in field-based sports (Ryan et al., 2014). Since groin injuries represent between 14% and 18% of the total injuries in elite soccer, the literature has suggested monitoring hip adductor strength as a strategy to identify possible risk factors (Hägglund et al., 2009).

Comparing the physical attributes of soccer players competing at different levels has already attracted some empirical research (Cometti et al., 2001; Śliwowski et al., 2021), since it allows coaches and sports trainers to assess performance potential and make informed decisions during the players’ recruitment process and strategic planning. Concerning isokinetic strength assessment, in a study conducted with 95 soccer players from the French Divisions (elite, sub-elite, and amateur), elite players presented significantly higher KF peak torque values than amateurs at all angular velocities tested (Cometti et al., 2001). In another study, elite players outperformed their non-elite counterparts in KF performance, but not KE (Śliwowski et al., 2021). On the other hand, assessments of hip adduction strength performance among players from different competitive levels have been conducted in American football (Prendergast et al., 2016) and field hockey (Beddows et al., 2020; Dominik et al., 2019), with no significant differences being located between the PL and NPL performance. However, research on this topic among soccer players is still lacking.

Thus, screening and monitoring players’ knee and hip muscle strength is of great importance for soccer coaches and trainers, allowing them to identify players at risk of injury and providing them with specific prevention programs. Besides, understanding the profiles of top-level players, particularly the relationship between the physical attributes and match demands, may provide crucial insights into the soccer’s long-term development process. Therefore, to contribute both to research and practice, the specific aims of this study were threefold: (1) to compare knee and hip muscle strength between professional players competing at different levels; (2) to compare strength performance according to the PL and the NPL; and (3) to compare knee and hip muscle strength performance at two different moments of the season. Based on previous literature, it was hypothesized that (1) elite players would perform better than their lower-division peers in strength assessments; (2) better performance levels would be attained with the PL compared to the NPL; and (3) there would be an increase in strength output during the season.

## Methods

### Participants

Thirty-three male professional soccer players participated in this study. Thirteen players were in the elite group (EG), competing in the First Portuguese Soccer League, while twenty players were in the sub-elite group (SEG), competing in the Fourth Portuguese Soccer League. Limb preference was defined as the leg with which a player kicked the ball (Hoffman et al., 1998). Twenty-four players had the right lower limb as the preferred one, while nine had the left limb as the preferred one.

### Measures

#### Body Composition

Body height was measured to the nearest 0.01 cm using a stadiometer (SECA 213, Hamburg, Germany). Body composition was evaluated using a hand-to-foot bioelectrical impedance analysis (InBody 770, Cerritos, CA, USA) during the early morning with participants in a fasted state. Participants were barefoot and wearing only their underwear. On the platform, their feet were placed on the defined spots, and their arms were placed nearly 45º from their trunk until the assessment was concluded.

#### Knee Muscle Strength

Knee muscle strength was measured using the Biodex System 4 Pro Dynamometer (Shirley, NY, USA). The isokinetic strength of knee extensors (KEs) and knee flexors (KFs) from the preferred leg (PL) and the non-preferred leg (NPL) was recorded at an angular velocity of 60º/s. Before data collection, a 5-min warm-up on a reclining bicycle (Technogym Xt Pro 600 Recline, Cesena, Italy) was performed with an effort varying from levels 4 to 5, and at a cadence ranging between 50 to 60 rotations per minute. After the warm-up, participants were seated on the dynamometer following the manufacturer's guidelines, adopting a standardized position of 85º hip flexion from the anatomical position. The lever arm of the dynamometer was aligned with the lateral epicondyle of the knee. At the same time, the trunk, the evaluated thigh, and the leg were stabilized with belts. The range of motion was defined considering participants’ maximum knee extension. Then, participants were asked to bend the knee until 90º of flexion. Individual calibration for gravity correction was performed at 30º of knee flexion (Osternig, 1986). During testing, participants were asked to keep their arms crossed with the hand on the opposite shoulder holding the belts (Baltzopoulos et al., 1991). Additionally, verbal support was given throughout the tests. To ensure the correct execution, three trials were allowed before testing started (Croix et al., 2017). Afterwards, five repetitions of concentric contraction efforts of knee flexion and knee extension were performed at 60º/s, with a 60-s interval after the sequence. The analysis included the values of peak torque (PT), peak torque/bodyweight (PT/BW), and the hamstring-to-quadriceps strength ratio (H:Q) for KEs and KFs for both legs. The H:Q conventional ratio was calculated by dividing the mean concentric KF PT by the mean KE concentric PT over the five repetitions (Pinto et al., 2018).

#### Hip Adductor Squeeze Strength Test

Isometric hip adduction strength was assessed during the squeeze strength test performed with a dynamometer (Smart Groin Trainer, NeuroExcellence, Portugal). Hips were positioned in 45º flexion with knees flexed to 90º (Delahunt et al., 2011). The dynamometer was placed between the knee, and participants were instructed to squeeze as hard as possible for three maximum contractions. Each contraction was held for five seconds, and three minutes of rest were given in between. Before testing, all participants performed a familiarization trial to ensure correct execution. From the three data collection trials, the best score was retained for further analysis.

### Design and Procedures

The study was conducted during the 2021/2022 season. Players were assessed one month after the season began (moment 1, M1) and five months after the first assessment (moment 2, M2). Testing sessions of the EG were performed for two days in the morning, while the SEG was evaluated for the next three consecutive days under the same conditions. All protocols were conducted in a physical performance laboratory by trained staff from the research team. The study protocol was approved by the Ethics Committee of the Faculty of Human Kinetics, University of Lisbon (approval code: CEIFMH N°34/2021; approval date: 6 July 2021), and followed the Declaration of Helsinki. Participation was voluntary, and informed consent was signed before data collection.

### Statistical Analysis

Data are reported as mean ± standard deviation (SD). Shapiro-Wilk tests were used to check the normality of the data. The differences between groups (elite vs. sub-elite) regarding age, body composition, and strength were analyzed using the Mann-Whitney U test. The Wilcoxon Signed Rank Test was conducted to verify differences between the performance of the PL and the NPL in each group and to compare the performance for each variable between M1 and M2. All analyses were performed using IBM SPSS Statistics software 28.0 (SPSS Inc., Chicago, IL, USA). The significance level was set at 0.05.

## Results

Table 1 resumes descriptive statistics and the comparison of results between groups for CA and body composition. The EG was significantly older than the SEG (U = 52.000, *p* ≤ 0.01, r = 0.50).

**Table 1:** Descriptive statistics and comparison between groups for age and body composition

Variable	Elite (n = 13)	Sub-elite (n = 20)	Comparison		
	Mean ± SD	Mean ± SD	U	*p*	r
CA (years)	25.2 ± 4.1	21.2 ± 1.5	52.000	≤ 0.01	0.50
Height (cm)	182.6 ± 6.3	179.0 ± 8.5	89.500	0.20	0.26
Body mass (kg)	77.6 ± 7.2	73.7 ± 10.0	95.000	0.23	0.22
Body fat (%)	12.2 ± 3.4	12.4 ± 2.7	155.000	0.81	0.16
Fat-free mass (kg)	68.1 ± 6.3	64.4 ± 7.8	89.500	0.16	0.26

*CA (chronological age); SD (standard deviation)*

Descriptive statistics for knee and hip muscle strength according to the PL and the NPL and the comparison results between groups are presented in Table 2. At M1, significant differences were observed between groups for KF PT/BW (PL: *p* ≤ 0.01, r = 0.39; and NPL: *p* ≤ 0.01, r = 0.51), the H:Q ratio (PL: *p* ≤ 0.01, r = 0.38; and NPL: *p* ≤ 0.05, r = 0.28), and squeeze strength (PL: *p* ≤ 0.01, r = 0.51; and NPL: *p* ≤ 0.01, r = 0.54). At M2, significant differences were found between groups for KE PT/BW (PL: *p* ≤ 0.01, r = 0.40; and NPL: *p* ≤ 0.01, r = 0.40), and KF PT/BW (PL: *p* ≤ 0.01, r = 0.55; and NPL: *p* ≤ 0.01, r = 0.38).

**Table 2:** Descriptive statistics for knee and hip muscle strength according to the PL and NPL, and results of the comparison between groups

Variable	Elite (n = 13)		Sub-elite (n = 20)	
	PL	NPL	PL	NPL
*Moment 1*				
KE PT (N•m)	195.3 ± 30.2	194.4 ± 23.7	179.5 ± 32.7	175.2 ± 34.4
KE PT/BW N•m/kg)	2.53 ± 0.43	2.51 ± 0.20	2.43 ± 0.23	2.37 ± 0.25
KF PT (N•m)	113.3 ± 15.0*	113.3 ± 17.2*	93.9 ± 22.5	94.0 ± 18.5
KF PT/BW N•m/kg)	1.47 ± 0.22*	1.47 ± 0.28*	1.27 ± 0.19	1.28 ± 0.17
H:Q ratio (%)	0.59 ± 0.07*	0.59 ± 0.09	0.52 ± 0.06	0.54 ± 0.05
Adductor squeeze strength (kg)	50.3 ± 12.1*	51.2 ± 12.4*	40.3 ± 7.8	40.7 ± 6.7
				
*Moment 2*				
KE PT (N•m)	257.7 ± 43.5*	257.6 ± 45.6*	222.7 ± 40.5	214.7 ± 41.4
KE PT/BW N•m/kg)	3.35 ± 0.40*	3.34 ± 0.45*	3.02 ± 0.40	2.91 ± 0.43
KF PT (N•m)	150.8 ± 22.8*	148.2 ± 17.9*	119.4 ± 29.2	116.9 ± 26.9
KF PT/BW N•m/kg)	1.96 ± 0.21*	1.93 ± 0.21*	1.61 ± 0.27	1.58 ± 0.25
H:Q ratio (%)	0.59 ± 0.06	0.59 ± 0.12	0.54 ± 0.08	0.55 ± 0.08
Adductor squeeze strength (kg)	43.3 ± 6.6	42.6 ± 7.9	43.3 ± 7.7	43.7 ± 6.8

PT (peak torque), PT/BW (peak torque normalized by bodyweight), H:Q (hamstrings:quadriceps ratio), * significant differences found between groups (elite vs. sub-elite)

**Figure 1:**
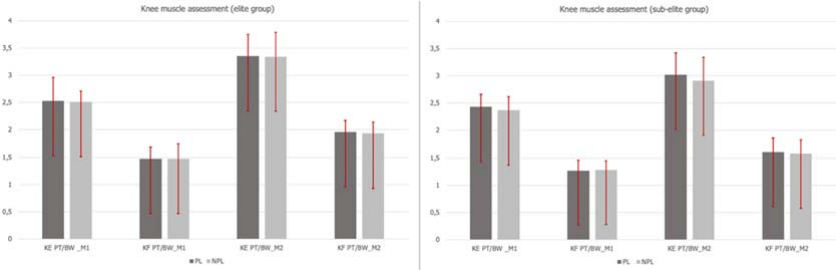
Knee muscle assessment according to the PL and the NPL.

**Figure 2:**
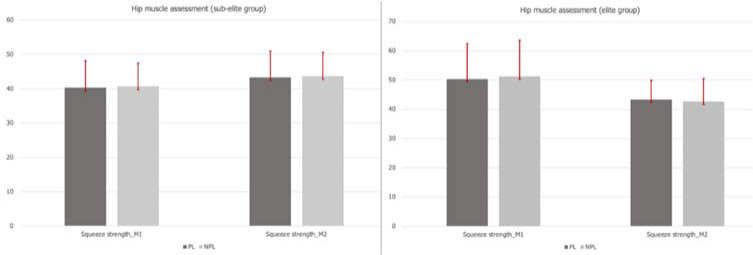
Hip muscle assessment according to the PL and the NPL.

Results of the comparison between the performance of the PL and the NPL are displayed in Figures 1 and 2 for knee and hip muscles, respectively. From M1 to M2, significant improvements were seen for KE (*p* ≤ 0.01) and KF (*p* ≤ 0.01) values in the PL and the NPL assessment in both groups. Regarding hip muscle strength, between assessments, a statistically significant decrease was observed in the EG (PL: *p* ≤ 0.05, NPL: *p* ≤ 0.05), while a significant increase was seen in the SEG (PL: *p* ≤ 0.05, NPL: *p* ≤ 0.01).

**Figure 3:**
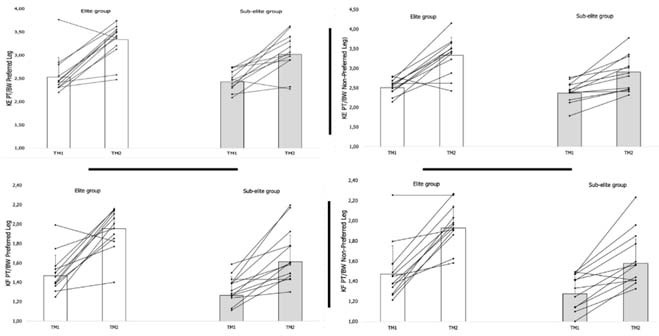
Comparison of the performance between M1 and M2 for knee muscle.

**Figure 4:**
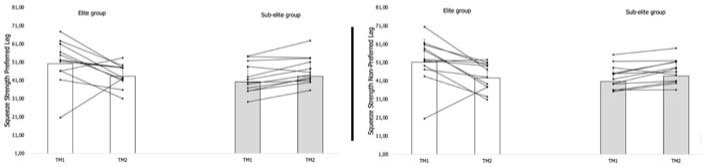
Comparison of the performance between M1 and M2 for hip muscle.

Finally, the results of the comparison between M1 and M2 for knee and hip muscle performance are presented in Figures 3 and 4. Significant increases were observed for KE PT/BW (PL: *p* ≤ 0.01, r = 0.52; and NPL: *p* ≤ 0.01, r = 0.53) and KF PT/BW (PL: *p* ≤ 0.01, r = 0.53; and NPL: *p* ≤ 0.01, r = 0.55) in the EG. In contrast, the squeeze strength performance of EG players was substantially lower at M2 compared to M1 (PL: *p* ≤ 0.01, r = 0.32; and NPL: *p* ≤ 0.01, r = 0.43). Among SEG players, significant increases were found in KE PT/BW (PL: *p* ≤ 0.01, r = 0.66; and NPL: *p* ≤ 0.01, r = 0.68) and KF PT/BW (PL: *p* ≤ 0.01, r = 0.68; and NPL: *p* ≤ 0.01, r = 0.65). The SEG also increased their squeeze strength from M1 to M2 (PL: *p* ≤ 0.01, r = 0.49; and NPL: *p* ≤ 0.01, r = 0.57).

## Discussion

This study assessed knee and hip muscle strength performance among professional soccer players competing at different levels. Overall, EG players outperformed SEG players in knee and hip muscle strength performance. No significant differences were found between the PL and the NPL in the variables assessed in both groups. From M1 to M2, significant improvements were observed in the EG for knee muscle strength, but not for hip muscle strength, while the SEG enhanced substantially their performance in both variables. To our knowledge, this is the first study with a detailed analysis of knee and hip muscle strength among professional male soccer players, confirming that top-level players tend to outperform their lower-division peers concerning strength indicators.

The analysis of physical attributes of individuals competing at different levels has shown significant differences between elite players and their lower-division peers in body composition (França et al., 2022; Mala et al., 2023), and several strength indicators (Cometti et al., 2001; França et al., 2022; Śliwowski et al., 2017). Regarding isokinetic assessment, KF PT and the H:Q ratio were significantly greater in international soccer players compared to non-international ones (Śliwowski et al., 2017). In a previous investigation, similar results were reported between professional soccer players and amateurs (Cometti et al., 2001). The results of the present study reinforce previous findings, underlining greater knee muscle strength of EG players compared to SEG players, not only in KFs (M1 and M2), but also in KEs (M2). Concerning hip muscle strength, significant differences were found in the squeeze strength test between groups exclusively at M1. Overall, data on comparing hip adduction performance among soccer players from different competitive levels are still scarce.

In the past, several researchers aimed to investigate the strength imbalance between the PL and the NPL in soccer (Daneshjoo et al., 2013; Rosa et al., 2022; Śliwowski et al., 2017). However, results on this matter are still controversial. Significant strength imbalances regarding knee function were reported in under-19 (Rosa et al., 2022) and elite male soccer players (Śliwowski et al., 2017). Consistently, mean values for the PL were statistically higher than the NPL, particularly concerning KFs (Rosa et al., 2022; Śliwowski et al., 2017). In contrast, although the PT values of the PL seemed higher than the NPL, no significant differences were observed among 26 young professional soccer players (Daneshjoo et al., 2013), which aligns with the present study results. Both groups presented superior mean values for knee muscle variables when performing with the PL at M1 and M2, but with no statistical difference. Although the sample of the present study was divided into two groups (EG and SEG), it should be underlined that all participants were professional soccer players. Therefore, players’ experience and training methods implemented at this level may justify the absence of significant strength imbalances.

On the other hand, past literature has described greater isometric hip adduction for the PL when compared to the NPL (Thorborg et al., 2011). In one study conducted with male soccer players aged 19.4 ± 1.5 years, the PL was 14% stronger than the NPL (Thorborg et al., 2011). In contrast, among 38 elite male American football players, authors reported no significant differences between the PL and the NPL in the hip adductor squeeze strength test (Lonie et al., 2020). In the present study, mean values of the PL and the NPL for the squeeze strength test followed different trends among groups. In the SEG, superior performance was seen with the NPL compared to the PL at both assessments. The EG performed better with the NPL at M1 and worse at M2. Even though, the overall analysis did not show significant strength imbalances. According to the literature, the players’ characteristics, particularly age and the experience level, could be related to strength imbalances. Players with long professional training experience tended to have lower strength imbalances than players with short professional training experience (Fousekis et al., 2010). The lack of substantial strength imbalances observed in the current study regarding hip adduction may reflect the players’ training experience and a balanced training regime.

Meanwhile, the EG and the SEG significantly improved KE and KF performance during the season, which is consistent with the results registered in under-19 players (Lehnert et al., 2014). However, contrary conclusions have been described among professional soccer players competing in the Turkish Super League. Authors reported no significant differences in isokinetic strength assessment at 60º/s from the beginning to the end of the season (Eniseler et al., 2012). Indeed, fluctuations in strength output may occur during the season influenced by training volume, recovery time, training adaptations, nutrition, and psychological factors (Hartmann et al., 2015; Turner and Stewart, 2014), which may justify the contradictory results from different investigations.

Concerning hip muscle strength, differences between groups in the squeeze strength test disappeared at M2. While SEG players enhanced significantly their performance, the opposite was observed in EG players. In a previous study on American football players, adductor squeeze strength was weaker at the end of the season than at the beginning (Lonie et al., 2020). Several factors (e.g., accumulated fatigue, training methods implemented, and players’ status) could impact these results which should be interpreted with caution. Although benefits in individual physical attributes should be expected from the training process, exposure to training and competition may result in fluctuations in strength output during the season.

Some limitations must be recognized in the current study. Firstly, the reduced sample size, particularly in the EG, limits the extension of these findings to other soccer populations with differing characteristics. Secondly, measuring PT at higher angular velocities might provide a more detailed assessment of knee function (Aagaard et al., 1995). Despite these limitations, this study provides novel and valuable insights into screening and monitoring players’ status. Identifying strength imbalances in knee and hip function may be crucial to estimating the likelihood of suffering an injury (Croisier et al., 2008; Hägglund et al., 2009). Therefore, seasonal monitoring for strength imbalances made by coaches and team trainers is valuable to identify at-risk players who could be engaged in specific prevention programs.

## Conclusions

The current study identified significantly better knee and hip muscle strength performance among top-level players than their lower-division peers. No substantial differences between PL and NPL performance were observed in knee function or hip adduction in both groups. During the season, players improved their KE and KF performance. However, only the SEG increased the performance in the squeeze strength test. In soccer, monitoring players' status according to strength imbalances between lower limbs may help identify players at risk of injury and frame them in specific prevention programs. The superior performance levels presented by elite players in knee and hip muscle strength reinforce strength as a crucial attribute to be promoted during the players’ long-term development.

## References

[ref1] Aagaard, P., Simonsen, E., Trolle, M., Bangsbo, J., & Klausen, K. (1995). Isokinetic hamstring/quadriceps strength ratio: influence from joint angular velocity, gravity correction and contraction mode. Acta Physiologica Scandinavica, 154(4), 421–427. 7484168 10.1111/j.1748-1716.1995.tb09927.x

[ref2] Baltzopoulos, V., Williams, J. G., & Brodie, D. A. (1991). Sources of error in isokinetic dynamometry: effects of visual feedback on maximum torque measurements. Journal of Orthopaedic & Sports Physical Therapy, 13(3), 138–142. 18796847 10.2519/jospt.1991.13.3.138

[ref3] Beddows, T. P., van Klij, P., Agricola, R., Tak, I. J., Piscaer, T., Verhaar, J. A., & Weir, A. (2020). Normal values for hip muscle strength and range of motion in elite, sub-elite and amateur male field hockey players. Physical Therapy in Sport, 46, 169–176. 32957033 10.1016/j.ptsp.2020.08.014

[ref4] Chena, M., Morcillo-Losa, J. A., Rodríguez-Hernández, M. L., Asín-Izquierdo, I., Pastora-Linares, B., & Zapardiel, J. C. (2022). Workloads of different soccer-specific drills in professional players. Journal of Human Kinetics, 84(1), 135–147. 36457458 10.2478/hukin-2022-000075 PMC9679172

[ref5] Cometti, G., Maffiuletti, N., Pousson, M., Chatard, J.-C., & Maffulli, N. (2001). Isokinetic strength and anaerobic power of elite, subelite and amateur French soccer players. International Journal of Sports Medicine, 22(01), 45–51. 11258641 10.1055/s-2001-11331

[ref6] Croisier, J.-L., Forthomme, B., Namurois, M.-H., Vanderthommen, M., & Crielaard, J.-M. (2002). Hamstring muscle strain recurrence and strength performance disorders. The American Journal of Sports Medicine 30(2), 199–203. 11912088 10.1177/03635465020300020901

[ref7] Croisier, J.-L., Ganteaume, S., Binet, J., Genty, M., & Ferret, J.-M. (2008). Strength imbalances and prevention of hamstring injury in professional soccer players: a prospective study. American Journal of Sports Medicine, 36(8), 1469–1475. 18448578 10.1177/0363546508316764

[ref8] Croix, M. D. S., ElNagar, Y. O., Iga, J., Ayala, F., & James, D. (2017). The impact of joint angle and movement velocity on sex differences in the functional hamstring/quadriceps ratio. The Knee, 24(4), 745–750. 28416087 10.1016/j.knee.2017.03.012

[ref9] Daneshjoo, A., Rahnama, N., Mokhtar, A. H., & Yusof, A. (2013). Bilateral and unilateral asymmetries of isokinetic strength and flexibility in male young professional soccer players. Journal of Human Kinetics, 36(1), 45–53. 23717354 10.2478/hukin-2013-0005 PMC3661893

[ref10] Delahunt, E., Kennelly, C., McEntee, B. L., Coughlan, G. F., & Green, B. S. (2011). The thigh adductor squeeze test: 45 of hip flexion as the optimal test position for eliciting adductor muscle activity and maximum pressure values. Manual Therapy, 16(5), 476–480. 21429785 10.1016/j.math.2011.02.014

[ref11] Dominik, N., Lipinska, P., Roczniok, R., Spieszny, M., & Stastny, P. (2019). Off-Ice Agility Provide Motor Transfer to On-Ice Skating Performance and Agility in Adolescent Ice Hockey Players. Journal of Sports Science & Medicine, 18(4), 680–694. www.jssm.org/jssm-18-680.xml>Fulltext 31827353 PMC6873137

[ref12] Engebretsen, A. H., Myklebust, G., Holme, I., Engebretsen, L., & Bahr, R. (2010). Intrinsic risk factors for groin injuries among male soccer players: a prospective cohort study. American Journal of Sports Medicine, 38(10), 2051–2057. 20699426 10.1177/0363546510375544

[ref13] Eniseler, N., Şahan, Ç., Vurgun, H., & Mavi, H. (2012). Isokinetic strength responses to season-long training and competition in Turkish elite soccer players. Journal of Human Kinetics, 31, 159–168. 23487507 10.2478/v10078-012-0017-5 PMC3588650

[ref14] Fernández-Baeza, D., Diaz-Ureña, G., & González-Millán, C. (2022). Differences in the Contractile Properties of the Biceps Femoris and Semitendinosus Muscles Throughout a Season in Professional Soccer Players. Journal of Human Kinetics, 84(1), 74–81. 36457475 10.2478/hukin-2022-0088 PMC9679184

[ref15] Fischerova, P., Nitychoruk, M., Smolka, W., Zak, M., Golas, A., & Maszczyk, A. (2021). The impact of strength training on the improvement of jumping ability and selected power parameters of the lower limbs in soccer players. Balt J Health Phys Activ, 13, 83-90. 10.29359/BJHPA.13.1.09

[ref16] Fousekis, K., Tsepis, E., & Vagenas, G. (2010). Lower limb strength in professional soccer players: profile, asymmetry, and training age. Journal of Sports Science & Medicine, 9(3), 364. 24149628 PMC3761700

[ref17] França, C., Ihle, A., Marques, A., Sarmento, H., Martins, F., Henriques, R., & Gouveia, É. R. (2022). Physical Development Differences between Professional Soccer Players from Different Competitive Levels. *Applied Sciences*, 12(14), 7343.

[ref18] Hägglund, M., Waldén, M., Bahr, R., & Ekstrand, J. (2005). Methods for epidemiological study of injuries to professional football players: developing the UEFA model. British Journal of Sports Medicine, 39(6), 340–346. 15911603 10.1136/bjsm.2005.018267 PMC1725241

[ref19] Hägglund, M., Waldén, M., & Ekstrand, J. (2009). Injuries among male and female elite football players. Scandinavian Journal of Medicine & Science in Sports, 19(6), 819–827. 18980604 10.1111/j.1600-0838.2008.00861.x

[ref20] Hartmann, H., Wirth, K., Keiner, M., Mickel, C., Sander, A., & Szilvas, E. (2015). Short-term periodization models: effects on strength and speed-strength performance. Sports Medicine, 45, 1373–1386. 26133514 10.1007/s40279-015-0355-2

[ref21] Hoffman, M., Schrader, J., Applegate, T., & Koceja, D. (1998). Unilateral postural control of the functionally dominant and nondominant extremities of healthy subjects. Journal of Athletic Training, 33(4), 319–322. 16558528 PMC1320581

[ref22] Konefał, M., Chmura, J., Zacharko, M., Zając, T., & Chmura, P. (2023). The Relationship among Acceleration, Deceleration and Changes of Direction in Repeated Small Sided Games. Journal of Human Kinetics, 85(1), 96–103. 36643839 10.2478/hukin-2022-0113 PMC9808809

[ref23] Krolikowska P, Golas A, Stastny P, Kokstejn J, Grzyb W, & Krzysztofik M. Abductor and adductor strength relation to sprint performance in soccer players. Balt J Health Phys Act. 2023;15(3):Article6. 10.29359/BJHPA.15.3.06

[ref24] Lehnert, M., Xaverová, Z., & Croix, M. D. S. (2014). Changes in muscle strength in U19 soccer players during an annual training cycle. Journal of Human Kinetics, 42(1), 175–185. 25414751 10.2478/hukin-2014-0072 PMC4234757

[ref25] Lonie, T. A., Brade, C. J., Finucane, M. E., Jacques, A., & Grisbrook, T. L. (2020). Hip adduction and abduction strength and adduction-to-abduction ratio changes across an Australian Football League season. Journal of Science and Medicine in Sport, 23(1), 2–6. 31445951 10.1016/j.jsams.2019.08.002

[ref26] López-Valenciano, A., Ruiz-Pérez, I., Garcia-Gómez, A., Vera-Garcia, F. J., Croix, M. D. S., Myer, G. D., & Ayala, F. (2020). Epidemiology of injuries in professional football: a systematic review and meta-analysis. British Journal of Sports Medicine, 54(12), 711–718. 31171515 10.1136/bjsports-2018-099577 PMC9929604

[ref27] Lord, C., Ma'ayah, F., & Blazevich, A. J. (2018). Change in knee flexor torque after fatiguing exercise identifies previous hamstring injury in football players. Scandinavian Journal of Medicine & Science in Sports, 28(3), 1235–1243. 29117428 10.1111/sms.13007

[ref28] Mala, L., Hank, M., Stastny, P., Zahalka, F., Ford, K., Zmijewski, P., Bujnovsky, D., Petr, M., & Maly, T. (2023). Elite young soccer players have smaller inter-limb asymmetry and better body composition than non-elite players. Biology of Sport, 40(1), 265–272. 36636184 10.5114/biolsport.2023.114840 PMC9806739

[ref29] Martins, F., França, C., Henriques, R., Ihle, A., Przednowek, K., Marques, A., Lopes, H., Sarmento, H., & Gouveia, É. R. (2022). Body composition variations between injured and non-injured professional soccer players. *Scientific Reports*, 12(1), 20779. 36456608 10.1038/s41598-022-24609-4 PMC9715542

[ref30] McBurnie, A. J., Harper, D. J., Jones, P. A., & Dos’ Santos, T. (2022). Deceleration training in team sports: Another potential ‘vaccine’ for sports-related injury? *Sports Medicine*, 1-12. 10.1007/s40279-021-01583-x PMC8761154 34716561

[ref31] Osternig, L. R. (1986). Isokinetic Dynamometry: Implications for Muscle Testing and Rehabilitation. Exercise and Sport Sciences Reviews, 14(1), 45–80. 3525192

[ref32] Pfirrmann, D., Herbst, M., Ingelfinger, P., Simon, P., & Tug, S. (2016). Analysis of injury incidences in male professional adult and elite youth soccer players: a systematic review. Journal of Athletic Training, 51(5), 410–424. 27244125 10.4085/1062-6050-51.6.03 PMC5013706

[ref33] Pinto, M. D., Blazevich, A. J., Andersen, L. L., Mil-Homens, P., & Pinto, R. S. (2018). Hamstring-to-quadriceps fatigue ratio offers new and different muscle function information than the conventional non-fatigued ratio. Scandinavian Journal of Medicine & Science in Sports, 28(1), 282–293. 28378509 10.1111/sms.12891

[ref34] Prendergast, N., Hopper, D., Finucane, M., & Grisbrook, T. L. (2016). Hip adduction and abduction strength profiles in elite, sub-elite and amateur Australian footballers. Journal of Science and Medicine in Sport, 19(9), 766–770. 26777723 10.1016/j.jsams.2015.12.005

[ref35] Rosa, F., Sarmento, H., Duarte, J. P., Barrera, J., Loureiro, F., Vaz, V., Saavedra, N., & Figueiredo, A. J. (2022). Knee and hip agonist-antagonist relationship in male under-19 soccer players. Plos One, 17(4), e0266881. 35427407 10.1371/journal.pone.0266881 PMC9012372

[ref36] Ryan, J., DeBurca, N., & Mc Creesh, K. (2014). Risk factors for groin/hip injuries in field-based sports: a systematic review. British Journal of Sports Medicine, 48(14), 1089–096. 24795341 10.1136/bjsports-2013-092263

[ref37] Śliwowski, R., Grygorowicz, M., Hojszyk, R., & Jadczak, Ł. (2017). The isokinetic strength profile of elite soccer players according to playing position. *Plos One*, 12(7), e0182177. 28759603 10.1371/journal.pone.0182177 PMC5536282

[ref38] Śliwowski, R., Marynowicz, J., Grygorowicz, M., Wieczorek, A., & Jadczak, Ł. (2021). Are there differences in concentric isokinetic strength performance profiles between international and non-international elite soccer players? International Journal of Environmental Research and Public Health, 18(1), 35. 10.3390/ijerph18010035 PMC7793063 33374580

[ref39] Stone, M. H., Moir, G., Glaister, M., & Sanders, R. (2002). How much strength is necessary? Physical Therapy in Sport, 3(2), 88–96.

[ref40] Suchomel, T. J., Nimphius, S., & Stone, M. H. (2016). The importance of muscular strength in athletic performance. Sports Medicine, 46, 1419–1449. 26838985 10.1007/s40279-016-0486-0

[ref41] Thorborg, K., Couppé, C., Petersen, J., Magnusson, S., & Hölmich, P. (2011). Eccentric hip adduction and abduction strength in elite soccer players and matched controls: a cross-sectional study. British Journal of Sports Medicine, 45(1), 10–13. 19850576 10.1136/bjsm.2009.061762

[ref42] Turner, A. N., & Stewart, P. F. (2014). Strength and conditioning for soccer players. Strength & Conditioning Journal, 36(4), 1–13.

